# Single-cell analysis reveals exosome-associated biomarkers for prognostic prediction and immunotherapy in lung adenocarcinoma

**DOI:** 10.18632/aging.205140

**Published:** 2023-10-24

**Authors:** Shengrong Lin, Shengjie Zhou, Xin Han, Yang Yang, Hao Zhou, Xuejiao Chang, Yefeng Zhou, Yuqin Ding, Huihui Lin, Qing Hu

**Affiliations:** 1 Department of Thoracic Surgery, Dongtai People’s Hospital, Dongtai 224299, China; 2 Department of Hematology, Dongtai People’s Hospital, Dongtai 224299, China

**Keywords:** lung adenocarcinoma, exosome, TME, immunotherapy, prognosis

## Abstract

Background: Exosomes play a crucial role in tumor initiation and progression, yet the precise involvement of exosome-related genes (ERGs) in lung adenocarcinoma (LUAD) remains unclear.

Methods: We conducted a comprehensive investigation of ERGs within the tumor microenvironment (TME) of LUAD using single-cell RNA sequencing (scRNA-seq) analysis. Multiple scoring methods were employed to assess exosome activity (EA). Differences in cell communication were examined between high and low EA groups, utilizing the “CellChat” R package. Subsequently, we leveraged multiple bulk RNA-seq datasets to develop and validate exosome-associated signatures (EAS), enabling a multifaceted exploration of prognosis and immunotherapy outcomes between high- and low-risk groups.

Results: In the LUAD TME, epithelial cells demonstrated the highest EA, with even more elevated levels observed in advanced LUAD epithelial cells. The high-EA group exhibited enhanced intercellular interactions. EAS were established through the analysis of multiple bulk RNA-seq datasets. Patients in the high-risk group exhibited poorer overall survival (OS), reduced immune infiltration, and decreased expression of immune checkpoint genes. Finally, we experimentally validated the high expression of SEC61G in LUAD cell lines and demonstrated that knockdown of SEC61G reduced the proliferative capacity of LUAD cells using colony formation assays.

Conclusion: The integration of single-cell and bulk RNA-seq analyses culminated in the development of the profound and significant EAS, which imparts invaluable insights for the clinical diagnosis and therapeutic management of LUAD patients.

## INTRODUCTION

Based on the most recent global cancer report released by the International Agency for Research on Cancer, it has been revealed that lung cancer (LC) is the second most frequently detected form of cancer and is the primary cause of cancer-related fatalities. The incidence and mortality rates of this disease are recorded at 11.4% and 18.0% respectively [1]. Immunotherapy, a groundbreaking treatment method, has brought about a significant transformation in the management of lung adenocarcinoma (LUAD), which constitutes approximately 40% of all histological types of LC. It has proven to be an effective therapeutic approach for various types of cancer [2]. However, only a minority of LC patients exhibit durable responses to immunotherapy. Therefore, the identification of reliable biomarkers is crucial for the implementation of immunotherapy and predicting the prognosis of LC patients [3–7].

Extracellular vesicles, ranging in size from 30 to 150 nm, are cell-derived vesicles that can transmit signaling molecules involved in cellular physiological regulation and participate in tumor invasion and metastasis [8]. Studies have revealed that extracellular vesicles promote tumor cells to evade immune surveillance and can serve as immunotherapeutic agents by altering the secretion of tumor-derived extracellular vesicles [9, 10].

Several studies have preliminarily elucidated the biological significance of extracellular vesicles in LC [11, 12]. Wang et al. discovered that extracellular vesicle miR-17-5p promotes osteoclastogenesis in LC by targeting PTEN and activating the PI3K/Akt pathway, thereby contributing to LC bone metastasis [13]. Li et al. found that hypoxia-induced extracellular vesicle miR-101, which is dependent on HIF1α, activates macrophages and induces inflammation in the tumor microenvironment (TME) [14]. Xue et al. identified extracellular vesicle miR-151a-5p, miR-10b-5p, miR-192-5p, miR-106b-3p, and miR-484 as potential prognostic markers in LUAD [15]. However, the roles and mechanisms of extracellular vesicle-associated genes in LC are still under investigation.

Single-cell sequencing technology, a novel sequencing technique, enables the measurement of the entire transcriptome at the single-cell resolution, allowing for the differentiation of different cell types. It can rapidly identify genetic differences between cancer and non-cancer cells, elucidate molecular mechanisms driving tumor development, and reveal somatic mutations during tumor evolution. By unraveling the heterogeneity of the TME, this method has been utilized to identify unique immune cell subpopulations potentially associated with tumor immune surveillance, thereby suggesting potential drug targets [16, 17]. Some studies have indicated that intra-tumoral heterogeneity contributes to cancer progression and enhances treatment resistance. Single-cell RNA sequencing (scRNA-seq) has been employed to assess the prognosis and drug resistance of LC, breast cancer, ovarian cancer, and gastric cancer [18, 19].

Therefore, establishing a signature based on extracellular vesicle-associated genes may serve as an effective approach for predicting the immunotherapeutic response and prognosis of tumor patients, which is also the objective of this study.

## METHODS

### Dataset source

Bulk RNA-seq data, mutation data, and clinical characteristics of patients diagnosed with LUAD were obtained from The Cancer Genome Atlas (TCGA) database (https://portal.gdc.cancer.gov/). The scRNA-seq dataset GSE131907 [20], which encompassed tissues from 20 LUAD patients, including 11 surgically resected tumor tissue samples, 4 biopsy samples obtained through puncture, and 5 pleural effusions, was sourced from the Gene Expression Omnibus (GEO) database (http://www.ncbi.nlm.nih.gov/geo/). Additionally, external validation cohorts (GSE30219, *n* = 86; GSE31210, *n* = 227; and GSE42127, *n* = 133) were retrieved from the GEO database. To ensure data comparability, the expression data were transformed into the transcripts per million (TPM) format. Addressing potential batch effects was accomplished using the “combat” function of the “sva” R package [21, 22]. Furthermore, the TCGA database standardized the data format by applying a log2 transformation to the bulk sequencing data, mutation data, and clinical details of LUAD patients before analysis.

### Single-cell dataset analysis

The R package “Seurat” [23–25] was employed for the processes of cell clustering and dimension reduction. In order to exclude specific cells, criteria were implemented, considering those that exhibited an expression of more than 6,000 or fewer than 300 genes, or a proportion of unique molecular identifiers (UMIs) derived from the mitochondrial genome that surpassed 10%. Through the application of principal component analysis (PCA) on the genes expressed with variability, the dataset’s dimensionality was effectively reduced. Subsequently, clustering analysis was conducted utilizing the “FindClusters” function, incorporating 20 PCA components and a resolution parameter of 1.2. Canonical marker genes were employed to annotate the resulting two-dimensional representation of cell clusters, thereby facilitating the identification of known biological cell types. The Seurat “FindAllMarkers” function was utilized to determine marker genes associated with cell clusters, making comparisons between cells within a specific cluster and those in all other clusters. The “cellchat” R package [26] was employed to infer communication networks between cell subpopulations. Scoring of exosome gene sets was carried out utilizing various methods such as “AUCell,” “UCell,” “Singscore,” “ssgsea,” and “AddModuleScore”.

### Building a high-performance EAS

Prognostic key genes were identified through the implementation of univariate Cox regression and lasso regression analyses [27, 28]. Subsequently, a refinement process was undertaken to select the genes and determine their corresponding coefficients, utilizing multivariate Cox regression [29, 30]. The calculation of the risk score for LUAD patients was performed using the following formula: The risk score was calculated as follows: Risk score = Σ [Coef (k) × Expr (k)], where Coef (k) represents the abbreviation for regression coefficients, and Expr (k) denotes the expression level of prognostic model genes. The application of the risk score calculation was applied to the dataset’s LUAD patients, leading to their stratification into high- and low-risk groups based on the median risk score. The model’s predictive performance was assessed through the utilization of receiver operating characteristic (ROC) curves, with exceptional performance indicated by area under the curve (AUC) values surpassing 0.65. PCA analysis was employed to visually depict the distribution of patients among different risk groups.

### Nomogram construction and evaluation

An enhanced and more precise nomogram was developed by merging the risk score with clinical characteristics, utilizing the “rms” R package [31]. This process significantly augmented the prognostic predictive ability. The efficacy of the nomogram was assessed through the utilization of the c-index and ROC curves. Stratified analyses based on age, pathological T, N, and clinical stage were performed to evaluate the predictive significance of both the risk score and clinical features.

### Enrichment analysis

In order to evaluate the biological characteristics, the utilization of Gene Set Variation Analysis (GSVA) and Gene Set Enrichment Analysis (GSEA) was implemented. For this analysis, downloadable files from the GSEA website were employed, specifically the files titled “h.all.v7.5.1.symbols.gmt,” “c5.go.v2023.1.Hs.symbols.gmt,” and “c5.go.v2023.1.Hs.symbols.gmt.” The quantification of enrichment scores for 29 immune signatures was performed using the ssGSEA approach.

### Mutations between different risk groups

The “maftools” R package [32] was utilized to conduct a comprehensive examination of somatic mutations in the high- and low-risk group of LUAD. The mutation annotation format (MAF) was generated from data extracted from the TCGA database. The assessment of tumor mutation burden (TMB) was performed for each patient with LUAD. The visualization of the mutation landscape and immune infiltration scores was achieved through the utilization of the “ComplexHeatmap” R package [33]. Based on the median risk score and median TMB, TCGA-LUAD patients were classified into four distinct groups, and a comparison was made between their survival disparities in relation to the median risk score and TMB.

### The TME and immunotherapy

The evaluation of immune cell content involved the utilization of seven immune infiltration algorithms, accessed through the TIMER 2.0 database (http://timer.comp-genomics.org/). Heatmaps were employed to visually depict the variations in immune cell infiltration across different risk groups. Furthermore, the “estimate” R package [34] was employed to calculate the immunological scores, stromal scores, and ESTIMATE scores of LUAD patients. In order to predict the responsiveness to immunotherapy, The Cancer Immunome Atlas (TCIA) database was explored for Immunophenoscores (IPS) associated with TCGA-LUAD. A comparison of IPS was performed between the high-risk and low-risk groups in this study [35]. Additionally, the “oncoPredict” R package was utilized to predict potentially effective chemotherapeutic agents between the risk groups [36].

### Cell lines culture and qRT-PCR

BEAS-2B cells, which are normal human lung epithelial cells, along with A549 and H1299 cells, representing human LUAD cell lines, were obtained from the Cell Resource Center of Shanghai Life Sciences Institute. These cells were cultured in F12K or RPMI-1640 supplemented with 10% fetal bovine serum (FBS), 1% streptomycin, and penicillin. The cell cultures were maintained at a temperature of 37°C, under conditions of 5% CO2 and 95% humidity. The extraction of total RNA from the cell lines was carried out following the manufacturer’s instructions using TRIzol. Subsequently, cDNA synthesis was performed utilizing the PrimeScriptTM RT kit. Real-time polymerase chain reaction (RT-PCR) was conducted using SYBR Green Master Mix, and the expression levels of each mRNA were normalized to the GAPDH mRNA level. Quantification of the expression levels was performed using the 2^−ΔΔCt^ method. The primers used for the experiment were provided by Tsingke Biotech (Beijing, China).

### Colony formation

A transfection of 1000 cells was performed, and they were subsequently placed in 6-well plates for approximately 14 days. After a two-week period, cell clones were visually observed without the aid of magnification. Following this, the cells were washed and fixed in 4% paraformaldehyde (PFA) for 15 minutes. Staining with crystal violet (Solarbio, China) was conducted for 20 minutes, followed by air drying at room temperature. The cell count per well was then determined.

### Statistical methods

The statistical analyses and data processing procedures were carried out using R, specifically version 4.2.0. To establish statistical significance, survival analysis was conducted using Kaplan-Meier curves, and the log-rank test was employed. All survival curves were generated using the “survminer” R package. Heatmaps, on the other hand, were generated using the “pheatmap” R package [37]. For variables demonstrating a normal distribution, quantitative differences were assessed through either a two-tailed *t*-test or a one-way analysis of variance (ANOVA). In cases where the data did not follow a normal distribution, the Wilcoxon test or Kruskal-Wallis test was utilized. All statistical analyses were conducted within the R environment, with a *P* < 0.05 considered as indicating statistical significance.

### Availability of supporting data

The datasets analyzed in the current study are available in the TCGA repository (http://cancergenome.nih.gov/), and GEO (https://www.ncbi.nlm.nih.gov/geo/).

## RESULTS

### The scRNA profiling of LUAD

**Figure 1:**
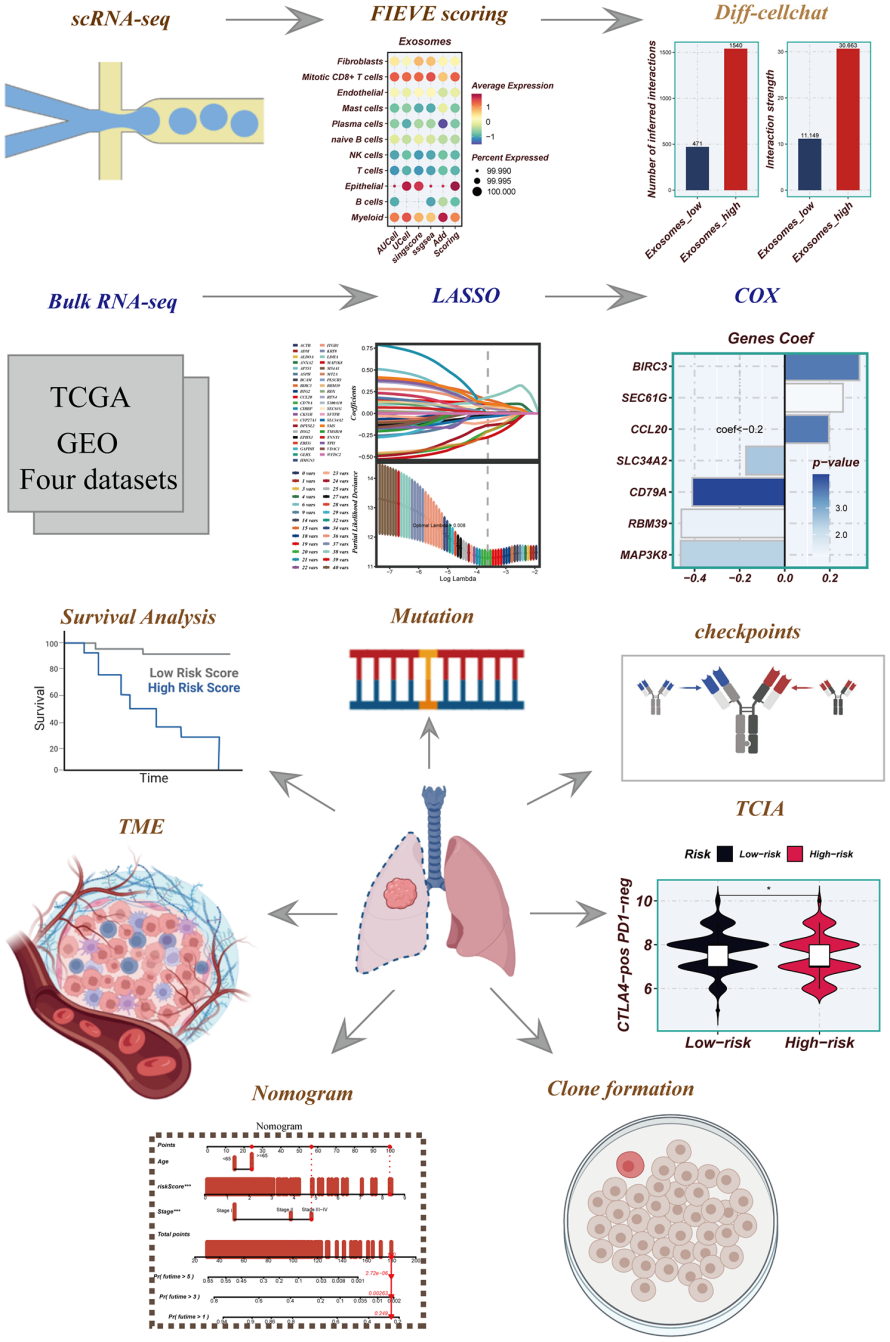
Flowchart for this study.

**Figure 2:**
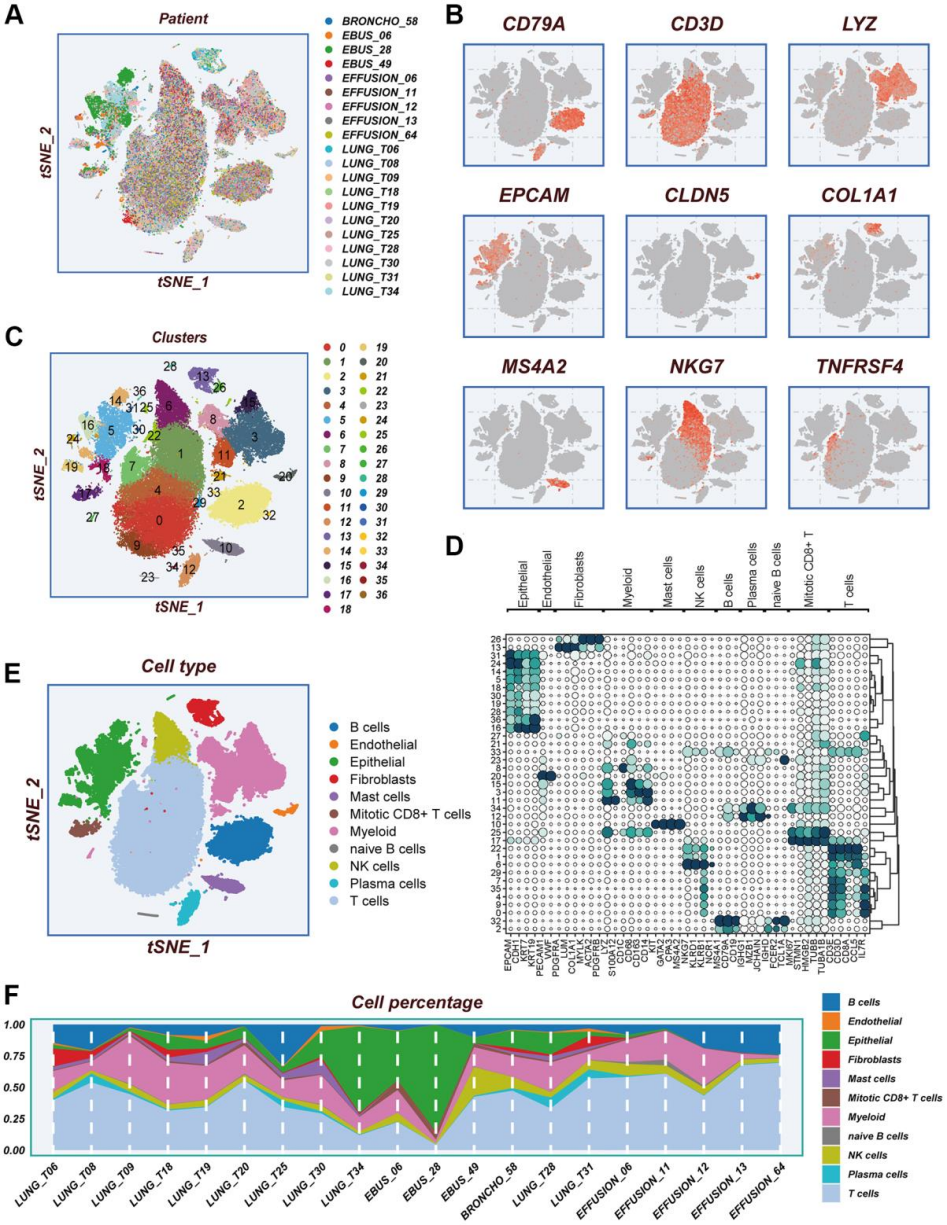
**Notes on cellular subpopulations.** (**A**) There was no significant batch effect on the cell distribution of the samples. (**B**) Show expression of typical cell type marker genes. (**C**) tSNE diagram of descending clustering binning. (**D**) A bubble chart showing the typical marker gene expression corresponding to each subgroup. (**E**) Cells are annotated into 11 different cell types. (**F**) The proportion of 11 cell types in different samples.

The study’s flow chart is presented in Figure 1. The scRNA-seq dataset underwent quality control measures. The expression characteristics displayed by each individual sample are illustrated in Supplementary Figure 1A, 1B. No significant fluctuations in cell cycles were observed in the principal component analysis (PCA) reduction plot, as depicted in Supplementary Figure 1C. A total of 20 samples were included in this study, and the cellular distribution remained relatively constant across each sample, suggesting minimal batch effects. Therefore, the samples were deemed suitable for subsequent analysis (Figure 2A). The expression of representative genes used for cell type identification is demonstrated in Figure 2B. By utilizing the tSNE dimensionality reduction algorithm, all cells were classified into 37 more detailed clusters (Figure 2C). The expression of characteristic marker genes corresponding to each cell cluster is visualized in the bubble plot shown in Figure 2D. The presence of 11 distinct cell types, such as fibroblasts, B cells, and NK cells, is revealed in Figure 2E. Furthermore, Figure 2F presents the proportional distribution of the 11 cell types in different samples.

### Exploring exosome activity within the single cell microenvironment

**Figure 3:**
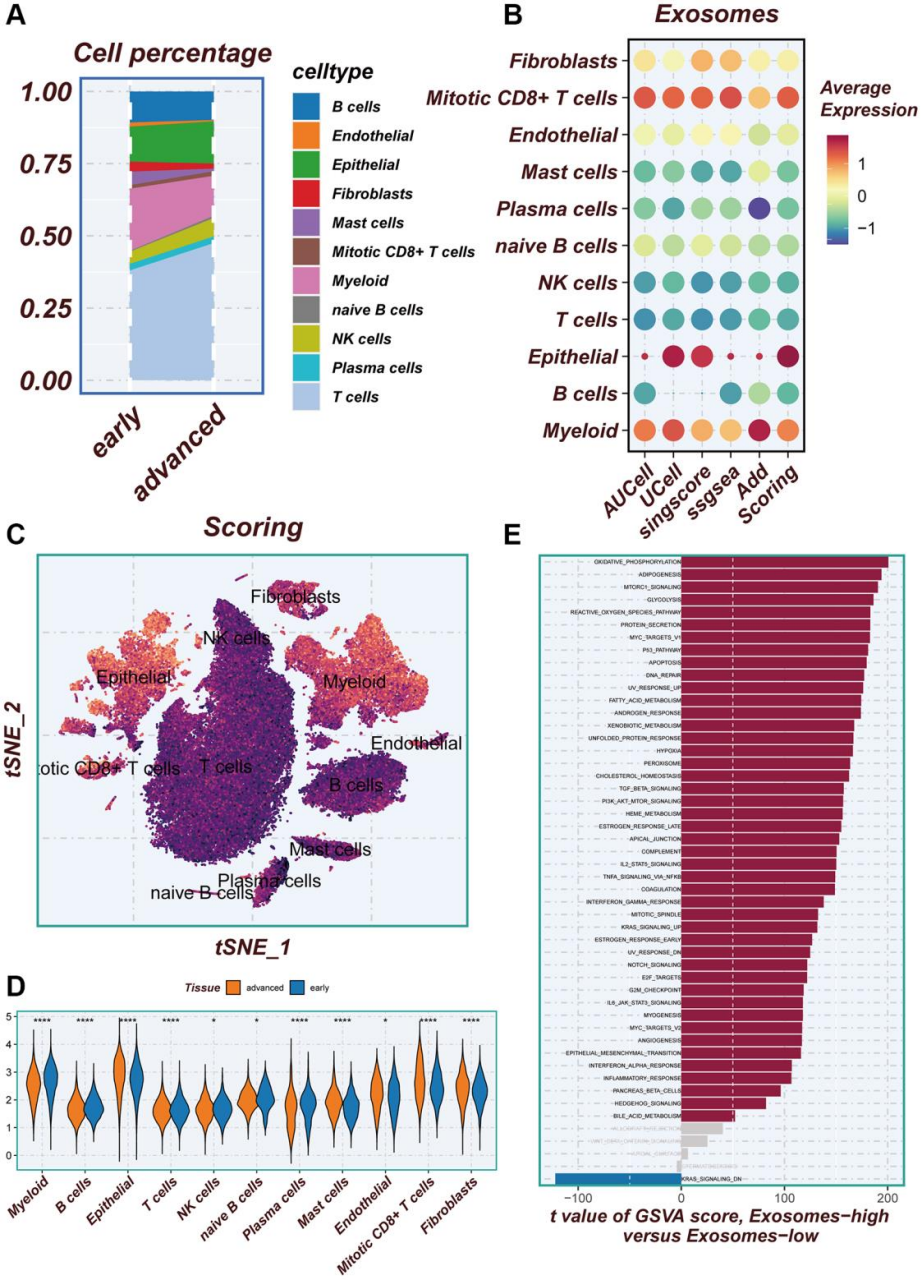
**Illustrating the identification of differentially expressed genes through AUCell method.** (**A**) Variations in the proportion of cells within the tissues of early-stage and advanced lung cancer were examined. (**B**) The average expression levels of ERGs were assessed in 11 cell types using five different scoring methods. (**C**) All cells were categorized into high- and low-groups based on their scores according to ERGs. (**D**) The levels of exosome-related genes between early-stage and advanced lung cancer tissues were compared. (**E**) The pathway of significant differences between the high- and low-groups of exosome levels was explored using hallmark gene sets.

There are differences in the percentage of cells between early-stage and advanced LUAD tissues (Figure 3A). In Figure 3B, a combination of five scoring methods (including AUCell, Ucell, singscore, ssgsea, and Addmodulescore) was employed to assess exosome activity (EA), revealing that epithelial cells exhibited the highest exosome activity. The tSNE diagram displayed the exosome activity across various cell types, highlighting stronger EA in epithelial and myeloid cells (Figure 3C). Figure 3D demonstrated significant disparities in exosomal activity levels between early-stage and advanced LUAD tissues. To unravel the underlying biological mechanisms associated with the different scoring scores, the hallmark gene set was utilized to explore the pathways that exhibited significant differences between the high- and low-EA groups. The principal enrichment pathways observed in the high-EA groups included oxidative phosphorylation, adipogenesis, and the p53 pathway (Figure 3E).

### Cellular interactions analysis

**Figure 4:**
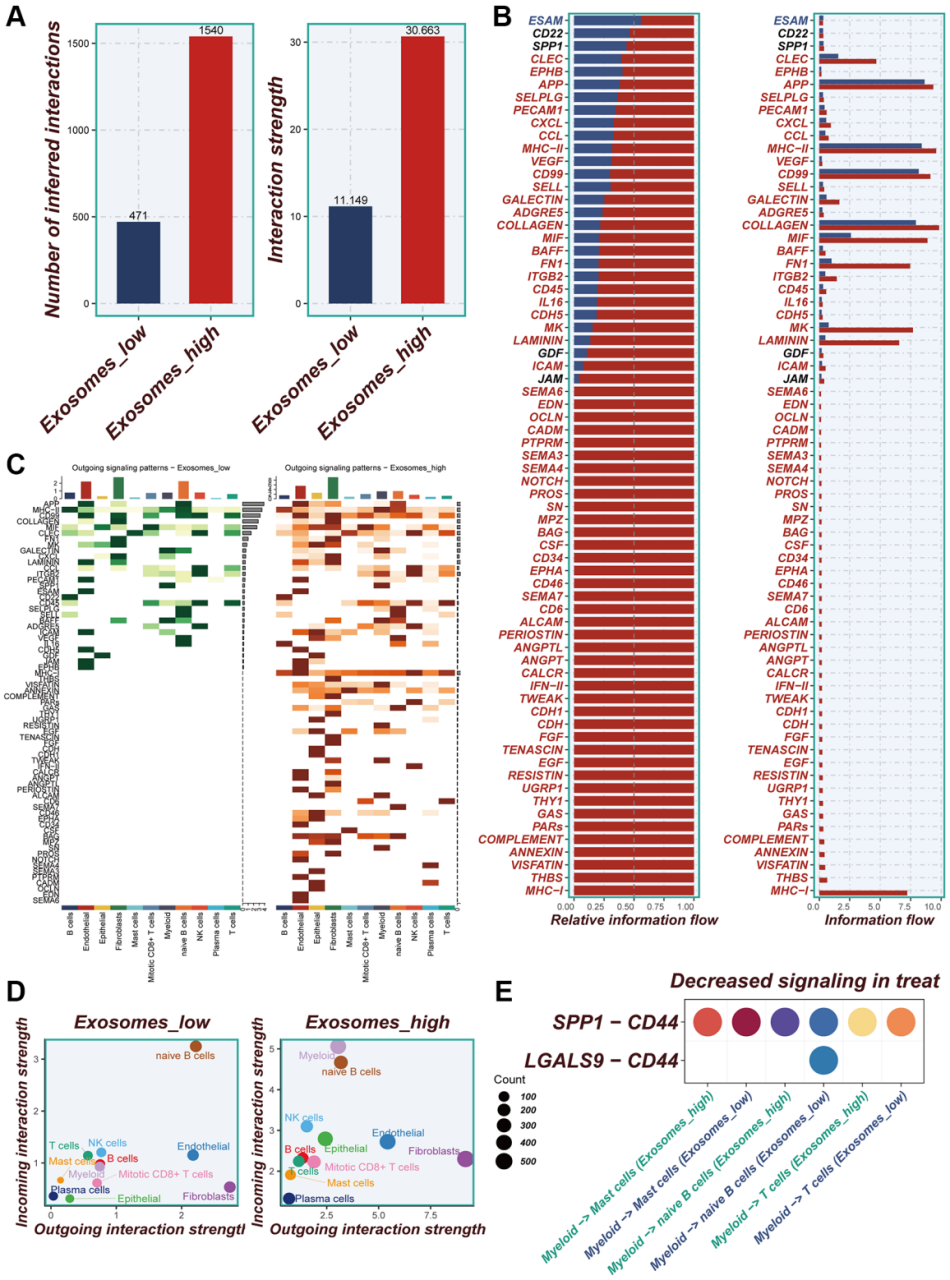
**Cellular interactions analysis.** (**A**) Differences in the number of cellular communications between groups with high and low expression of EA. (**B**) The number and percentage of various signaling pathways in the high-risk and low-risk groups. (**C**) Heatmaps demonstrating the strength of outgoing signaling pathways in different cell subpopulations. (**D**) A scatter plot showing the distribution of different cell populations in the intensity of outgoing and incoming signaling interactions. (**E**) Expression of ligand-receptor pair genes in cell populations.

Differences in the number of cellular communications between groups with high- and low-EA groups were presented in Figure 4A and Supplementary Figure 2A, 2B. Figure 4B showed the number and percentage of various signaling pathways in the high- and low-EA. Significant differences in signals emitted between the high- and low-EA groups, with more signals active only in the high-EA group (Figure 4C). Significant alterations were also observed in the roles fulfilled by various cell types within different subgroups. In the low-EA group, both myeloid and epithelial cells exhibited weak efferent and afferent signals. However, in the high- EA group, their signaling capabilities were significantly enhanced (Figure 4D). Supplementary Figure 2C and Figure 4E unveiled significant discrepancies in the profiles of ligand-receptor pairs between the high- and low-EA groups. Notably, the SPP1-CD44 receptor-ligand pair emerged as a more crucial player in the low-EA group.

### Construction of a risk model

**Figure 5:**
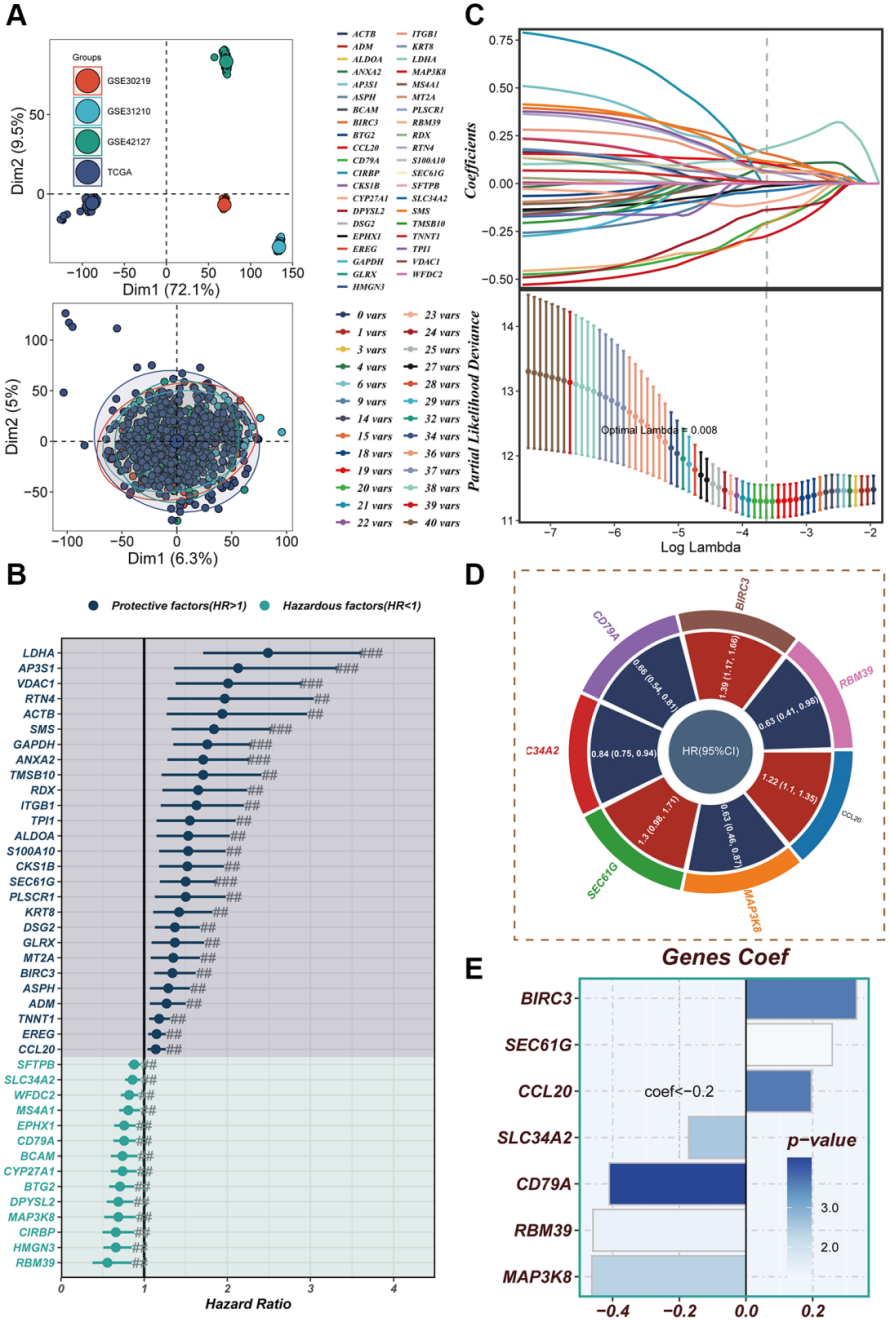
**Construction of a risk model.** (**A**) In both the TCGA cohort and GEO cohort, no significant batch effect was observed, and batch effect removal was performed. (**B**) Significant variables affecting prognosis were screened using LASSO regression. (**C**) The results of univariate COX analysis were presented in a forest plot. (**D**) Genes included in the risk model after multivariate regression analysis were illustrated in a circle plot. (**E**) The distribution of coefficient values of model genes was displayed.

In Figure 5A, the TCGA and GEO independent cohorts were observed to exhibit significant batch effects. However, after removing the batch effect, more accurate results were obtained. The training set from TCGA was utilized for model construction, leading to the identification of 41 prognostic genes through univariate COX analysis (*P* < 0.01). The forest plot depicted the results of the univariate COX analysis, revealing 14 hazardous factors and 27 protective factors (Figure 5B). Subsequently, LASSO and Cox regression analysis were employed to establish the prognostic model (Figure 5C). The hazard ratio (HR) values associated with each variable included in the model were presented in Figure 5D, while Figure 5E displayed the corresponding coefficients of specific variables.

### Evaluation of the model

**Figure 6:**
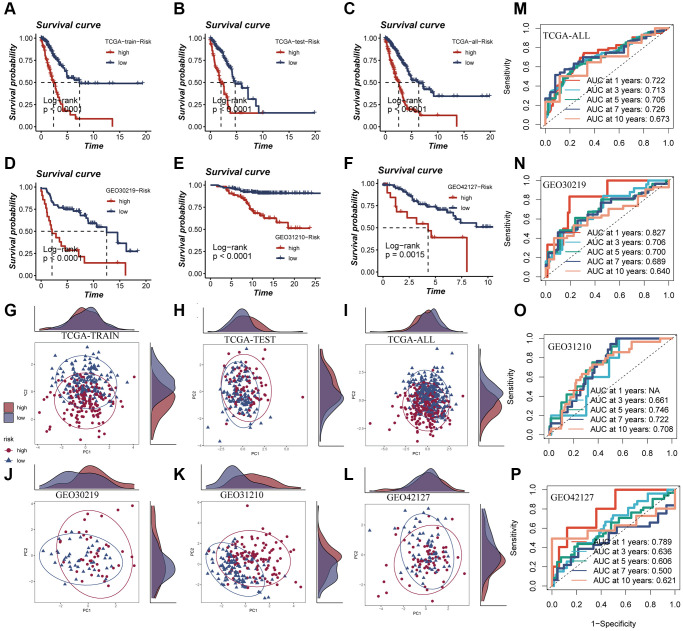
**Survival curves, sample distribution and ROC curves of high- and low-risk groups.** (**A**–**F**) The survival differences of different risk groups in TCGA-TRAIN, TCGA-TEST, TCGA-ALL, GEO30219, GEO30210 and GEO42127, respectively, were presented. (**G**–**L**) The PCA sample distribution of different risk groups in TCGA-TRAIN, TCGA-TEST, TCGA-ALL, GEO30219, GEO30210 and GEO42127, respectively, were presented. (**M**–**P**) The ROC curves of different risk groups in TCGA-TRAIN, GEO30219, GEO30210 and GEO42127 at 1-, 3-, 5-, 7-, and 10-years, respectively, were presented.

In Figure 6A–6C, it was observed that a worse prognosis was exhibited by the high-risk group in the TCGA training set, test set, and the entire cohort (*P* < 0.001). Additionally, a significantly poorer prognosis of patients in the high-risk group compared to the low-risk group was noted in the GEO30219 test cohort, GEO30210 test cohort, and GEO42127 test cohort (*P* < 0.001, Figure 6D–6F). These findings indicate that the prognostic model incorporating ERGs is highly accurate in predicting patient prognosis in both TCGA and GEO datasets. The effectiveness of the model in classifying LUAD patients into training and testing groups was demonstrated through PCA evaluation of the model for each of the seven genes in the TCGA training set, test set, and the entire cohort (Figure 6G–6I). Similar results were observed in the PCA assessment of the GEO30219 test cohort, GEO30210 test cohort, and GEO42127 test cohort (Figure 6J–6L). To further evaluate the accuracy of exosomes in assessing the prognosis of LUAD patients, ROC curve analysis was performed for the entire TCGA cohort, GEO30219 trial cohort, GEO30210 trial cohort, and GEO42127 trial cohort. The results demonstrated that good predictive performance was exhibited by the majority of the ROC curves (Figure 6M–6P).

### Clinical correlation and nomogram construction

**Figure 7:**
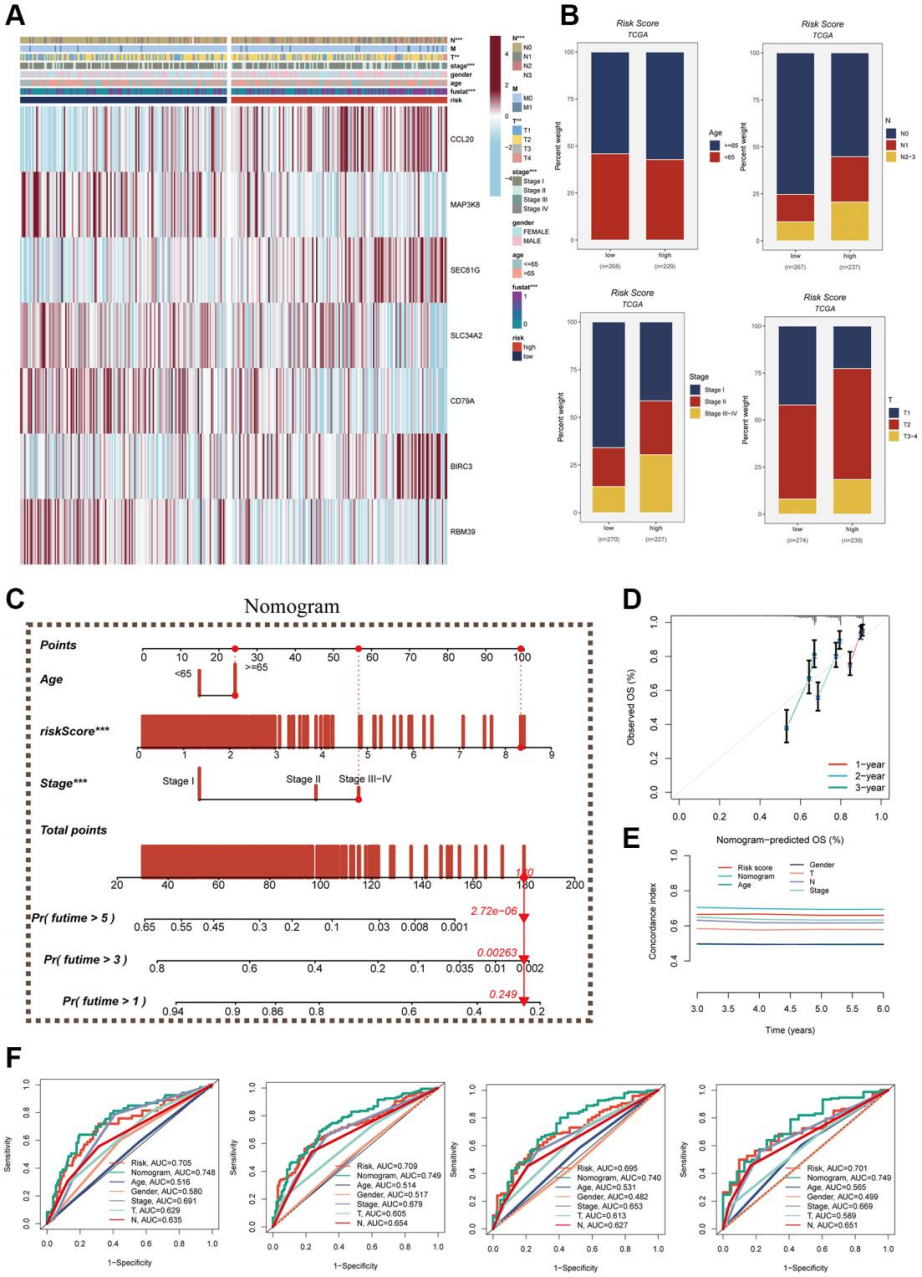
**Clinical correlation analysis and construction of nomogram.** (**A**) Heat map was constructed by combining clinical features and model gene expression to demonstrate the distribution of clinical features and model genes in high- and low-risk groups. (**B**) Bar graphs showing the proportion of T-stage, N-stage, fustat, and clinical stage in the high- and low-risk groups. (**C**) A nomogram was constructed by combining age, risk score and clinical stage. (**D**) Concordance index curves. (**E**) Decision curve. (**F**) ROC curves showing AUC values for clinical characteristics, risk scores and nomogram scores at 1-, 3-, 5-, and 7-years, respectively.

A heatmap was generated by combining clinical information and the high- and low-risk groups to visualize the distribution of clinical characteristics among different risk groups. Statistical analysis in Figure 7A revealed significant differences between the two groups concerning T and N stages, clinical stage, and fustat (*P* < 0.05). Notably, the high-risk group displayed a higher proportion of older patients and more advanced N and T stages (Figure 7B). Furthermore, a nomogram was constructed using clinical characteristics and risk scores (Figure 7C) to enhance the accuracy of prognosis prediction in LUAD patients. The nomogram plots can assist clinicians in assessing patient risk more accurately and guiding future treatment decisions. The calibration curve and decision curve analyses demonstrated the superior efficacy of this nomogram compared to other clinical indicators in predicting patient prognosis, thus serving as a valuable clinical decision-making tool (Figure 7D, 7E). Additionally, a comprehensive prognosis ROC analysis (Figure 7F) was conducted to evaluate the accuracy of the nomogram. The results exhibited area under the curve (AUC) values of 0.705, 0.709, 0.696, and 0.701 at 1, 3, 5, and 7 years, respectively.

### Enrichment analysis

**Figure 8:**
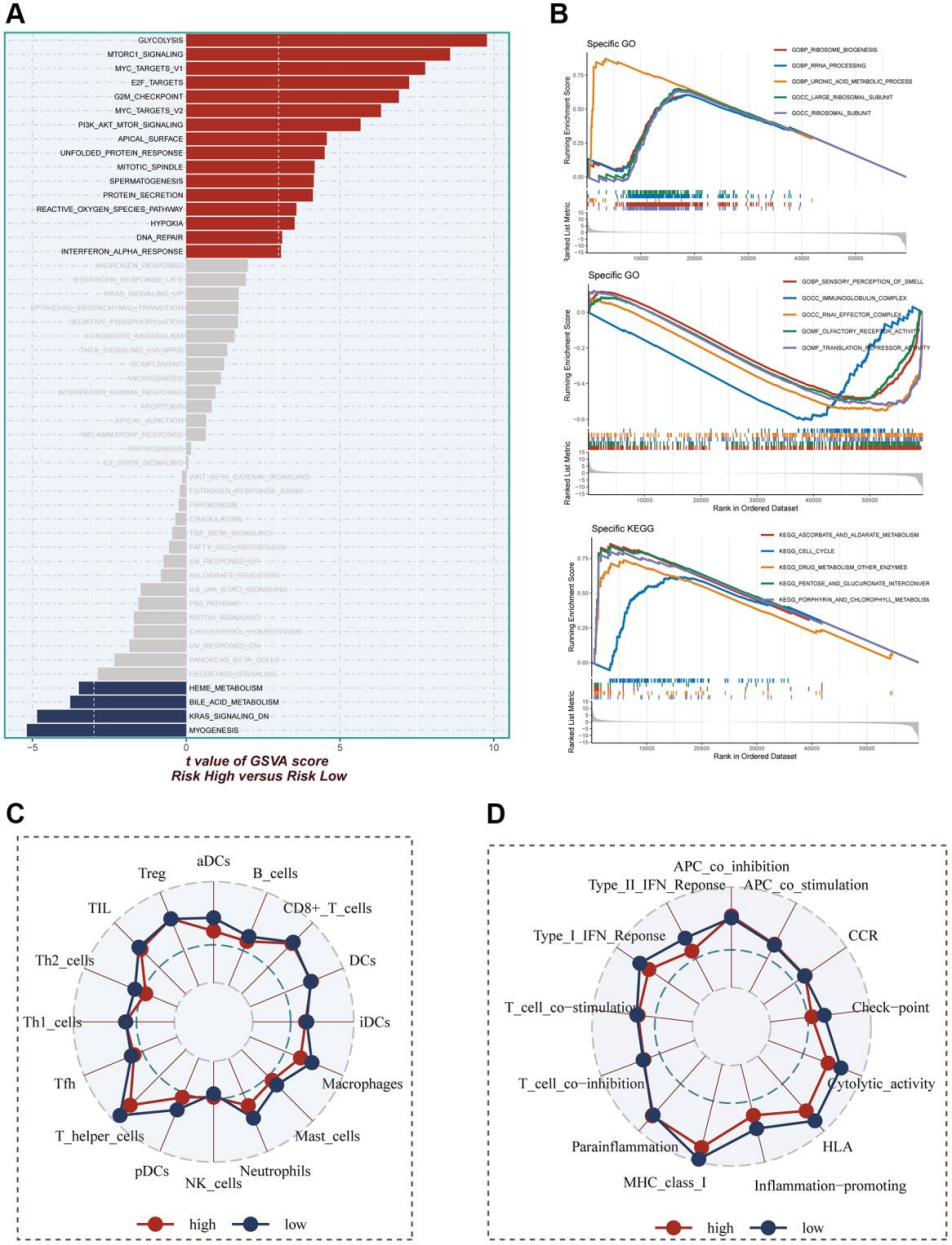
**Enrichment pathways between different risk groups.** (**A**) GSVA enrichment analysis demonstrates the enrichment of hallmark gene sets between different risk groups. (**B**) GSEA enrichment analysis demonstrating the enrichment of differential genes to GO pathways between high- and low-risk groups. (**C**, **D**) ssGSEA enrichment analysis demonstrating the enrichment of immune cell infiltration and immune-related pathways between high- and low-risk groups.

The evaluation of pathways exhibiting significant differences between the high- and low-risk groups was carried out using the hallmark gene set. In Figure 8A, it was demonstrated that enrichment in cell cycle-related pathways, including mTORC1 signaling, MYC targets V1, E2F targets, G2M checkpoint, and MYC targets V2, among others, was predominantly observed in the high-risk group. For GO and KEGG enrichment analysis, GSEA was employed. The GO enrichment results, as depicted in Figure 8B, indicated that the high-risk group exhibited significant enrichment in pathways such as ribosome biogenesis, rRNA processing, uronic acid metabolic process, and more. Conversely, the low-risk group primarily showed enrichment in pathways related to immunoglobulin complex and translation repressor activity. In terms of KEGG enrichment, the main pathways enriched in the high-risk group were cell cycle and pentose and glucuronate interconversion. To assess the differences in immune cell infiltration and immune-related pathways between the high- and low-risk groups, the ssGSEA method was utilized. The analysis revealed that the low-risk group exhibited higher levels of immune cell infiltration, including T helper cells, pDCs, macrophages, and others. Moreover, greater activity in certain immune-related pathways, such as Type II IFN response, checkpoint, HLA, among others, was demonstrated by the low-risk group (Figure 8C, 8D).

### Immune infiltration assessment and mutation landscape

**Figure 9:**
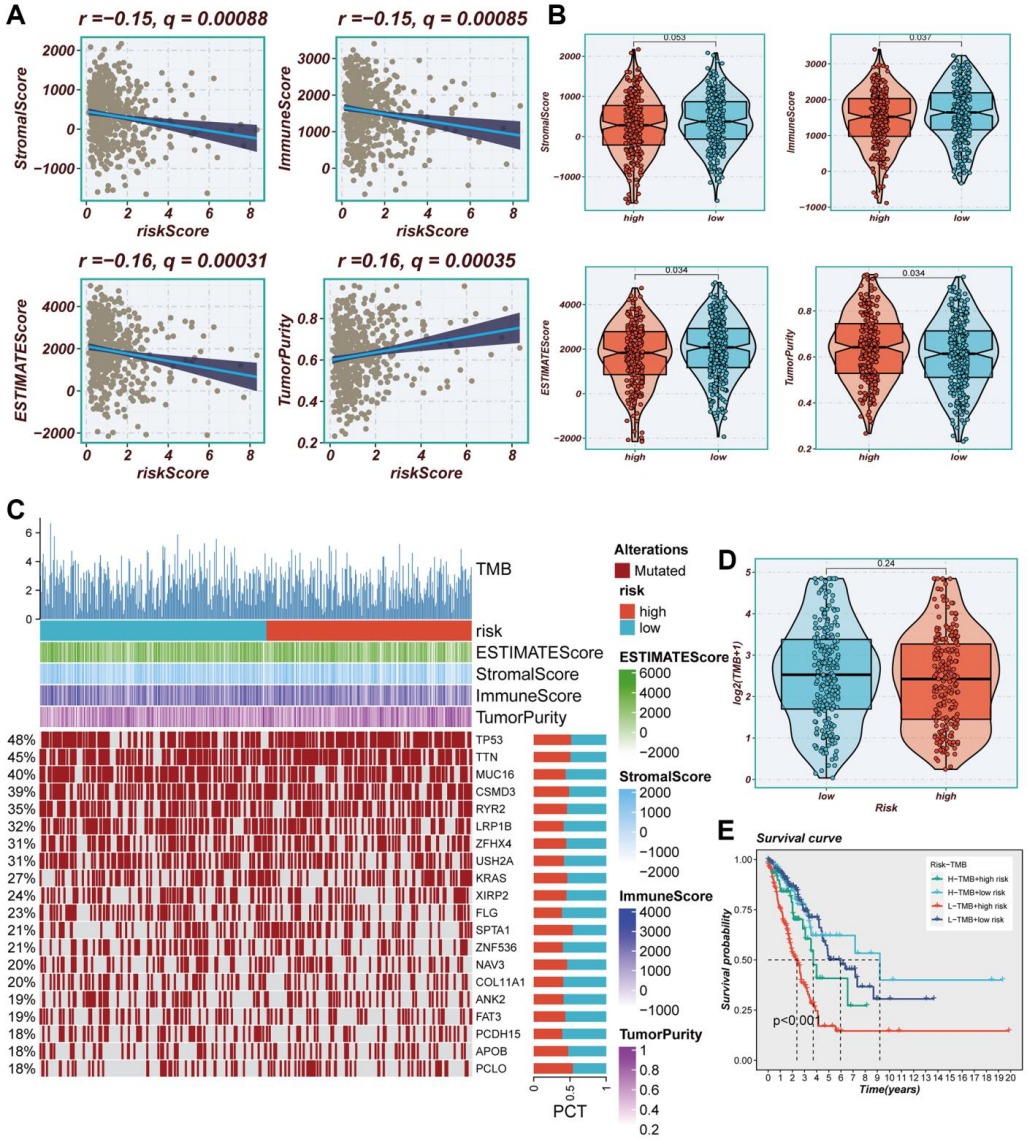
**Immune infiltration assessment.** (**A**) Scatter plot of correlation between risk score and stromal score, immune score, ESTIMATE score, and tumor purity. (**B**) Boxplots of differences between risk groups in stromal score, immune score, ESTIMATE score and tumor purity. (**C**) Heat map demonstrating the differences in immune cell infiltration between high- and low-risk groups assessed using four algorithms. (**D**) Boxplots of differences between risk groups in TMB. (**E**) Survival curves showing the difference between survival among four subgroups (high-risk and high-mutation, high-risk and low-mutation, low-risk and high-mutation, low-risk and low-mutation).

The degree of immune infiltration was evaluated using seven algorithms within the TIMER 2.0 database, and the comparison revealed greater immune cell infiltration within the low-risk group (Supplementary Figure 3). Immune infiltration levels were assessed using the “ESTIMATE” R package, wherein correlation analysis unveiled a noteworthy negative correlation between the risk score and immune score, alongside a positive correlation with tumor purity (Figure 9A). Figure 9B exhibited higher immune scores and ESTIMATE scores within the low-risk group (*P* < 0.05), indicating a heightened overall state of immunity and immunogenicity within low-risk group. Representative gene variants were compared between the high-risk and low-risk groups (Figure 9C). The top five genes in terms of mutation frequencies were TP53, TTN, MUC16, CSMD3, and RYR2. The low-risk group exhibited a higher TMB relative to the high-risk group (Figure 9D), albeit lacking statistical significance. Patients were stratified based on risk scores and TMB, revealing that the low-TMB and high-risk groups exhibited the most unfavorable prognosis (Figure 9E).

### Immunotherapy and chemotherapy drugs

**Figure 10:**
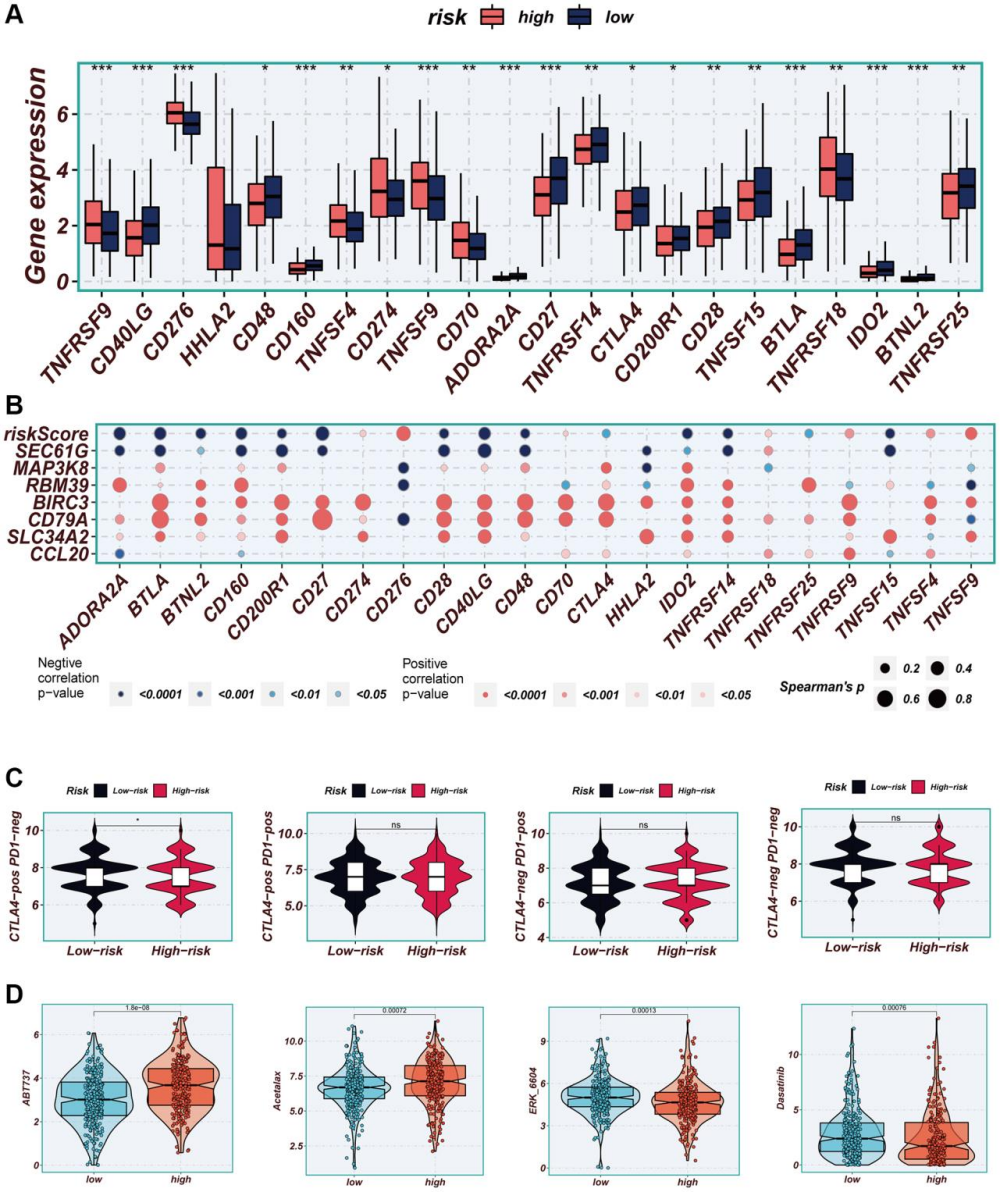
**Immune checkpoint and immunotherapy analysis.** (**A**) Boxplots showing the difference in immune checkpoint expression between high- and low-risk groups. (**B**) Correlation scatter plots showing the correlation between model genes and risk scores and immune checkpoint expression. (**C**) TCIA analysis showing the difference in IPS scores between different risk groups to infer the possible benefit of receiving PD-1 and CTLA-4 treatment in different risk groups. (**D**) Boxplots demonstrating the possible sensitivity of chemotherapeutic agents between different risk groups.

Considering the significance of immune checkpoints in the success of tumor immunotherapy, we investigated the differential expression of immune checkpoints between the two risk groups. Low-risk patients exhibited significant upregulation of thirteen immune checkpoint genes, including CD40LG, CD48, and CD27. In the high-risk group, seven immune checkpoint genes, including CD276, CD274, and CD70, were significantly elevated (Figure 10A). Correlation analysis, depicted in Figure 10B, illustrated the relationship between risk scores, model genes, and immune checkpoint gene expression. Red color indicated a positive correlation, while blue color indicated a negative correlation. It was evident that the risk score exhibited a significant negative correlation with the majority of immune checkpoint genes, such as BTLA, CD27, and CD48. Immunophenoscore (IPS) was employed to select patients likely to respond to immune therapy. In our study, we observed that low-risk patients had a higher IPS when receiving CTLA-4 immunotherapy (Figure 10C). This finding suggested that low-risk patients may demonstrate enhanced responsiveness to immune checkpoint inhibitors (ICIs) and derive greater benefits. By utilizing the “oncopredict” R package, we explored potentially effective chemotherapy drugs for both high- and low-risk groups. Our findings indicated that ABT737 and Acetalax may be more efficacious in low-risk patients, while ERK_6604 and Dasatinib may exhibit higher sensitivity in high-risk patients (Figure 10D).

### Experimental validation

**Figure 11:**
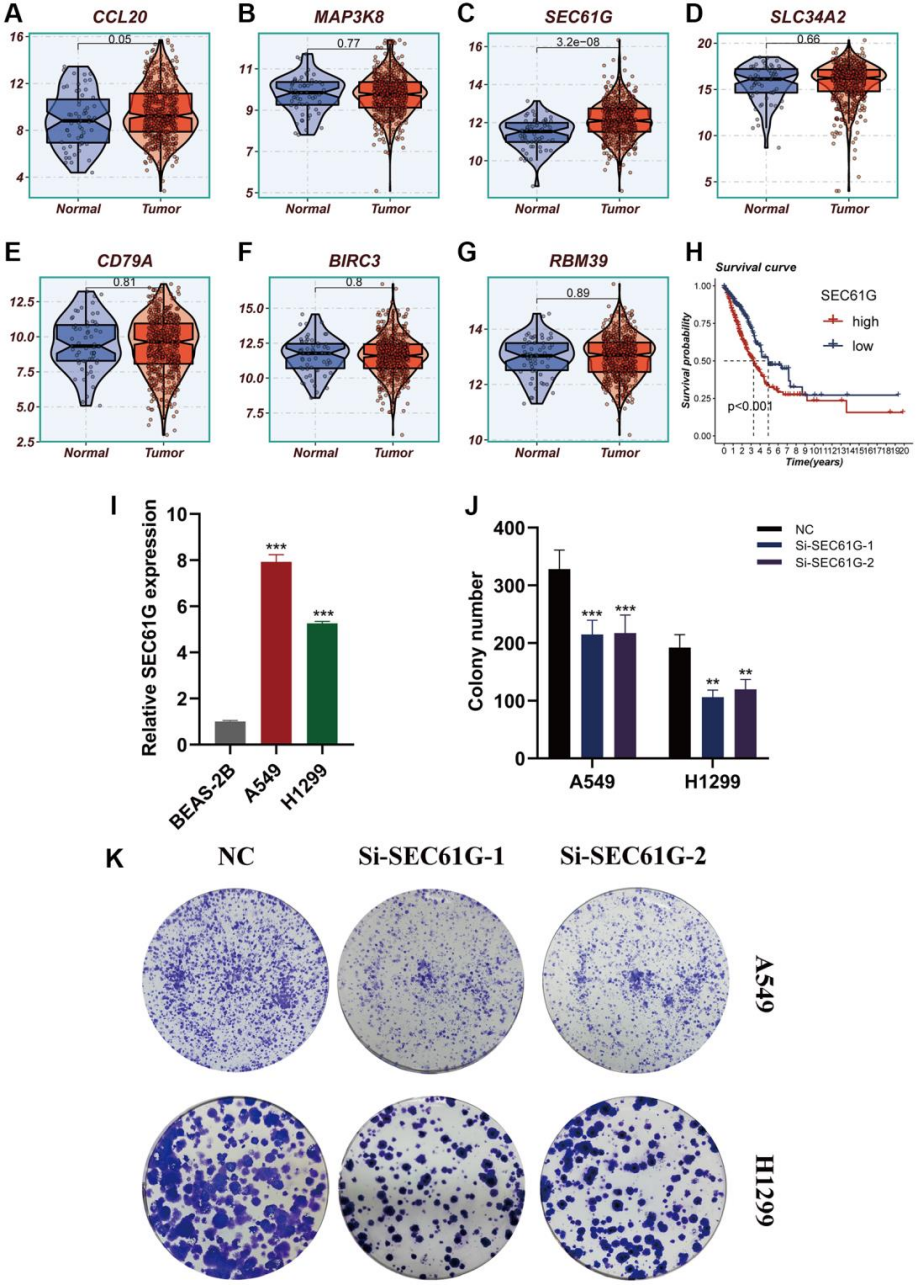
**Experimental validation of model gene and *in vitro* experiment with SEC61G knockdown.** (**A**–**G**) Boxplots showing the differential expression of CCL20, MAP3K8, SEC61G, SLC34A2, CD79A, BIRC3, and RBM39 between tumor and normal tissues. (**H**) Survival curves showing the difference between SEC61G high and low expression groups. (**I**) Histogram shows the relative SEC61G expression between BEAS-2B, A549 and H1299. (**J**, **K**) After SEC61G knockdown, the cloning ability of A549 and H1299 cell lines decreased significantly.

The expression differences of seven model genes were compared between tumor tissues and normal tissues, as depicted in Figure 11A–11G. Notably, high expression of SEC61G was observed in tumor tissues. Furthermore, Figure 11H illustrated that LUAD patients with high expression of SEC61G exhibited poorer survival outcomes. Additionally, the experiments demonstrated that A549 and H1299 LUAD cells exhibited higher expression of SEC61G compared to normal lung cells (Figure 11I). Downregulation of SEC61G resulted in a significant reduction in the number of cell clones within the LUAD cell lines (Figure 11J, 11K). These findings strongly suggest that high expression of SEC61G can promote the proliferation of LUAD cells.

## DISCUSSION

With an estimated 1.8 million deaths, accounting for approximately 18.0% of total cancer-related deaths, LC remains the primary contributor to cancer-related mortality. In terms of incidence, LC ranks second at 11.4%, following breast cancer. In 2020, LC stood as the second most frequently diagnosed cancer and a significant cause of cancer-related fatalities. It constituted around 11.4% of all diagnosed cancers and approximately 18.0% of all cancer-related deaths [1]. It is estimated that from 2020 to 2050, the macroeconomic cost of global cancer will reach 25.2 trillion US dollars, with the highest economic burden caused by tracheal, bronchus, and LC (15.4%, 3.9 trillion US dollars) [38]. LC patients are typically diagnosed at an advanced stage and can undergo surgical resection or chemotherapy; however, the treatment outcomes are often suboptimal. Immunotherapy is an innovative approach in cancer treatment, offering advantages that traditional anti-cancer therapies cannot match [3]. It can prolong progression-free survival (PFS) and OS by dynamically modulating the immune system to target cancer cells from multiple angles and directions, thereby helping the immune system to impede or slow down the growth of cancer cells, destroy cancer cells, or prevent cancer from spreading to other parts of the body [5, 39, 40]. However, immunotherapy also comes with complexities and uncertainties. Excessive activation of the immune system may lead to severe adverse reactions during treatment [17]. To enhance the effectiveness of immunotherapy and minimize the occurrence of adverse reactions, there is an urgent need to identify more accurate predictive indicators.

Exosomes, small vesicles released by cells carrying a diverse range of biologically active molecules derived from living cells, are known as extracellular vesicles. They can be taken up by adjacent cells through direct fusion, endocytosis, or specific receptor binding, thereby transferring the encapsulated information to target cells. In the context of the TME, exosomes serve as crucial regulatory factors in intercellular communication. They participate in cell-cell contacts and control cellular signal transduction, thus playing important roles in tumor development and progression. The significant association between exosomes and LC has been highlighted in numerous studies. Furthermore, exosomes can be detected in various body fluids, making them promising candidates as diagnostic and prognostic biomarkers for LC. In a study conducted by Grimolizzi et al., the levels of miR-126 were compared in serum, exosomes, and exosome-depleted serum of healthy individuals, as well as early and advanced non-small cell lung cancer (NSCLC) patients. It was found that miR-126 was uniformly distributed in healthy individuals, whereas in early and advanced NSCLC patients, miR-126 was primarily present in exosomes. These findings suggest the involvement of miR-126 in regulating the microenvironmental niche of NSCLC and highlight its potential value for NSCLC diagnosis and personalized therapy [4]. Elevated expression levels of exosomal miR-23b-3p, miR-10b-5p, and miR-21-5p were found to be associated with poor overall survival (OS) in LC patients, as reported by Liu et al. These findings suggest that plasma exosomal miR-23b-3p, miR-10b-5p, and miR-21-5p have potential as non-invasive prognostic biomarkers for LC [41]. In the study conducted by Kanaoka et al., a significant correlation was observed between exosomal miR-451a and lymph node metastasis, vascular invasion, and tumor stage in LC. It may serve as a reliable biomarker for predicting recurrence and prognosis in patients with stage I, II, and III non-small cell LC [42].

The objective of this study was to examine the association between ERGs and the prognosis of LUAD. Through COX regression and Lasso regression analyses, a prognostic model was developed utilizing seven ERGs. Based on the median risk value, patients were classified into high-risk and low-risk groups using the established model. Notably, the high-risk group demonstrated a notably inferior prognosis in comparison to the low-risk group. To validate the accuracy of the model, ROC curves were performed on the training cohort and testing cohorts. The AUC values of the TCGA cohort and the GEO30219 validation cohort were above 0.7 at 1 year, 3 years, and 5 years, indicating good discriminative ability. Although the AUC values of the GEO30210 and GEO42127 validation cohorts were slightly lower, they still demonstrated reasonable discriminative capacity. Furthermore, clinically relevant ROC curves and decision curves revealed that the risk score outperformed other clinical features in terms of clinical utility. Compared to the low-risk group, the high-risk group had a higher proportion of patients in stages II-IV, consistent with traditional clinical staging. These findings suggest that the model can provide more accurate prognostic predictions for LC patients.

Previous studies have indicated that patients with higher TMB may exhibit increased sensitivity to immunotherapy [15]. In our study, although the difference was not statistically significant, we observed that the low-risk group had higher TMB levels compared to the high-risk group. Further survival analysis revealed that patients in the high-risk group with low TMB had the poorest prognosis, suggesting that these patients may demonstrate better sensitivity to immunotherapy. Within the signature we developed, the gene SEC61G was associated with adverse prognosis in LUAD patients. Our cell experiments demonstrated elevated expression of SEC61G in LUAD tissues, and knockdown of SEC61G significantly decreased the proliferative capacity of LUAD cells. These findings provide additional evidence for the involvement of SEC61G in LUAD. A critical role in various tumors is played by SEC61G, which is a subunit of the endoplasmic reticulum translocon. In their study, Ma et al. observed high-expression SEC61G in breast cancer, which correlated with unfavorable prognosis. Furthermore, they demonstrated that overexpression of SEC61G contributes to the development and metastasis of breast cancer by modulating glycolysis, a process regulated by the transcription factor E2F1. These findings highlight the potential of targeting SEC61G as a therapeutic strategy for breast cancer treatment [43]. In the study conducted by Meng et al., the role of SEC61G in kidney cancer was explored, revealing its upregulation in tumor tissues and its correlation with unfavorable prognosis. Furthermore, the knockdown of SEC61G was observed to hinder cell proliferation, migration, and invasion, while promoting apoptosis. These findings suggest that SEC61G holds promise as both a potential prognostic biomarker and therapeutic target for kidney cancer [44]. Similarly, in our study, we identified SEC61G as a potential target for LUAD, further emphasizing its significance in cancer research.

Additional experimental validation is essential to confirm these findings, as the constructed EAS in this study enables the prediction of prognosis in patients with LUAD and reveals potential opportunities for the implementation of immunotherapy.

## Supplementary Materials

Supplementary Figures
