# Supplementary material for: Single-cell analysis reveals exosome-associated biomarkers for prognostic prediction and immunotherapy in lung adenocarcinoma
Source: Aging (Albany NY). 2023 Oct 24;15(20):11508–31. doi: 10.18632/aging.205140 (PMC10637798; doi:10.18632/aging.205140)
Supplement: Supplementary Figures [file aging-15-205140-s001.pdf]

SUPPLEMENTARY FIGURES

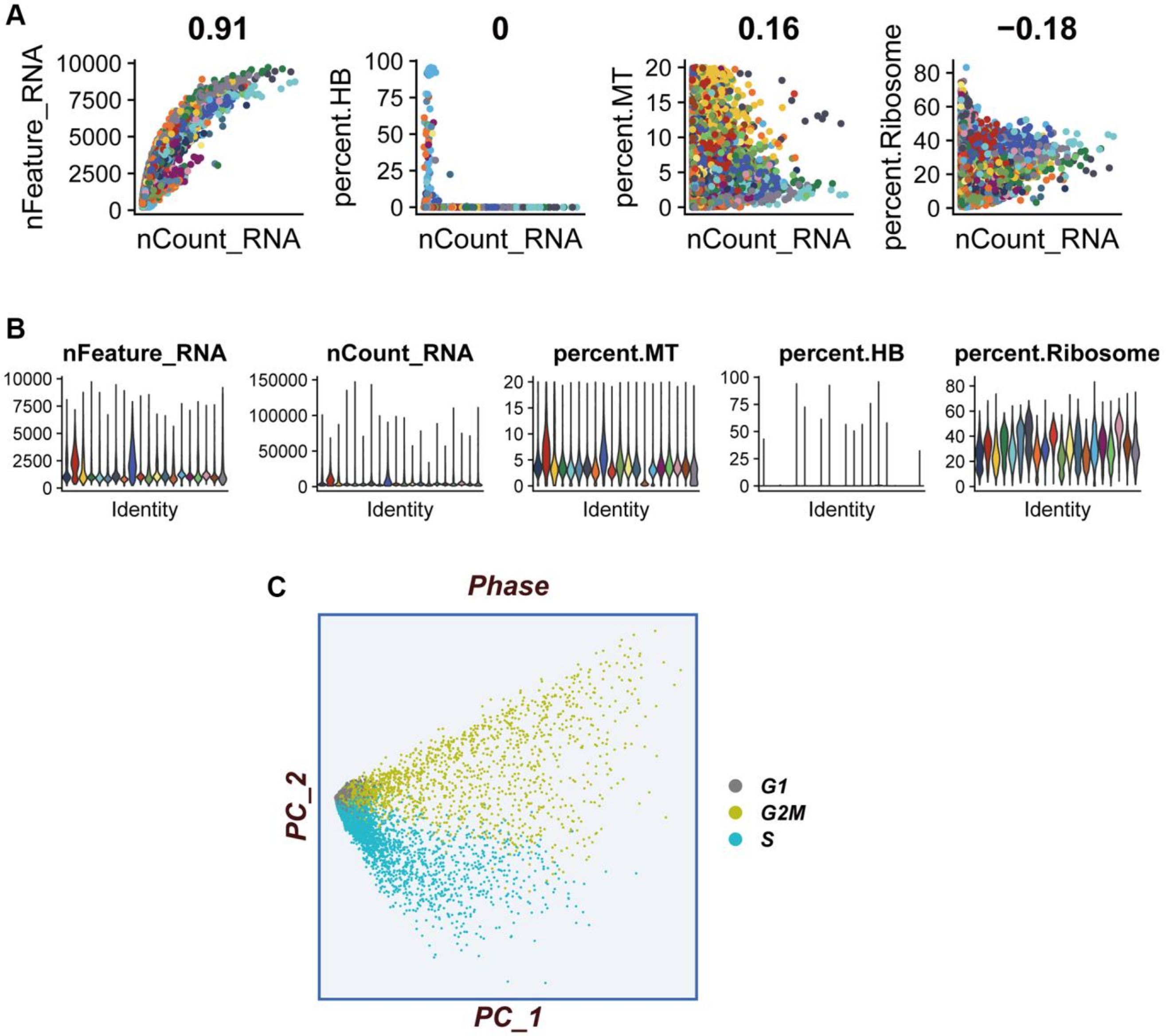

Supplementary Figure 1. Quality control and dimensionality reduction analysis of single cell sequencing data. (**A**) Sequencing depth is significantly and positively correlated with total intracellular sequence (R = 0.91). (**B**) Removal of non-compliant cells by restricting the ratio of mitochondrial genes, hemoglobin genes and ribosomal genes. (**C**) PCA analysis did not show any significant cell cycle changes.

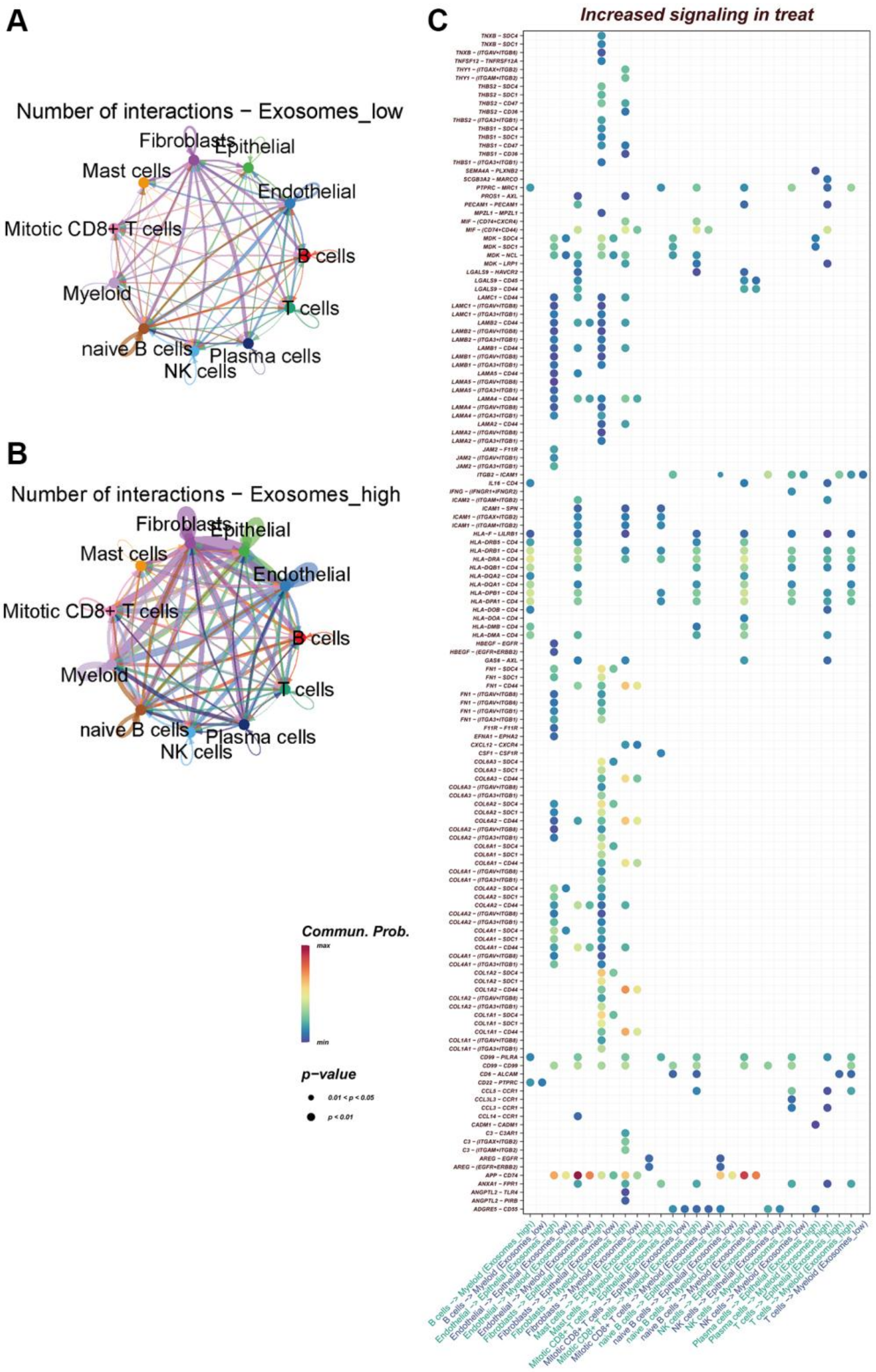

Supplementary Figure 2. Cell-to-cell communication. (**A**, **B**) Differences in the number of cellular communications between groups with high and low expression of EA. (**C**) Differential expression of ligand-receiving pairs between the high- and low-EA expression groups.

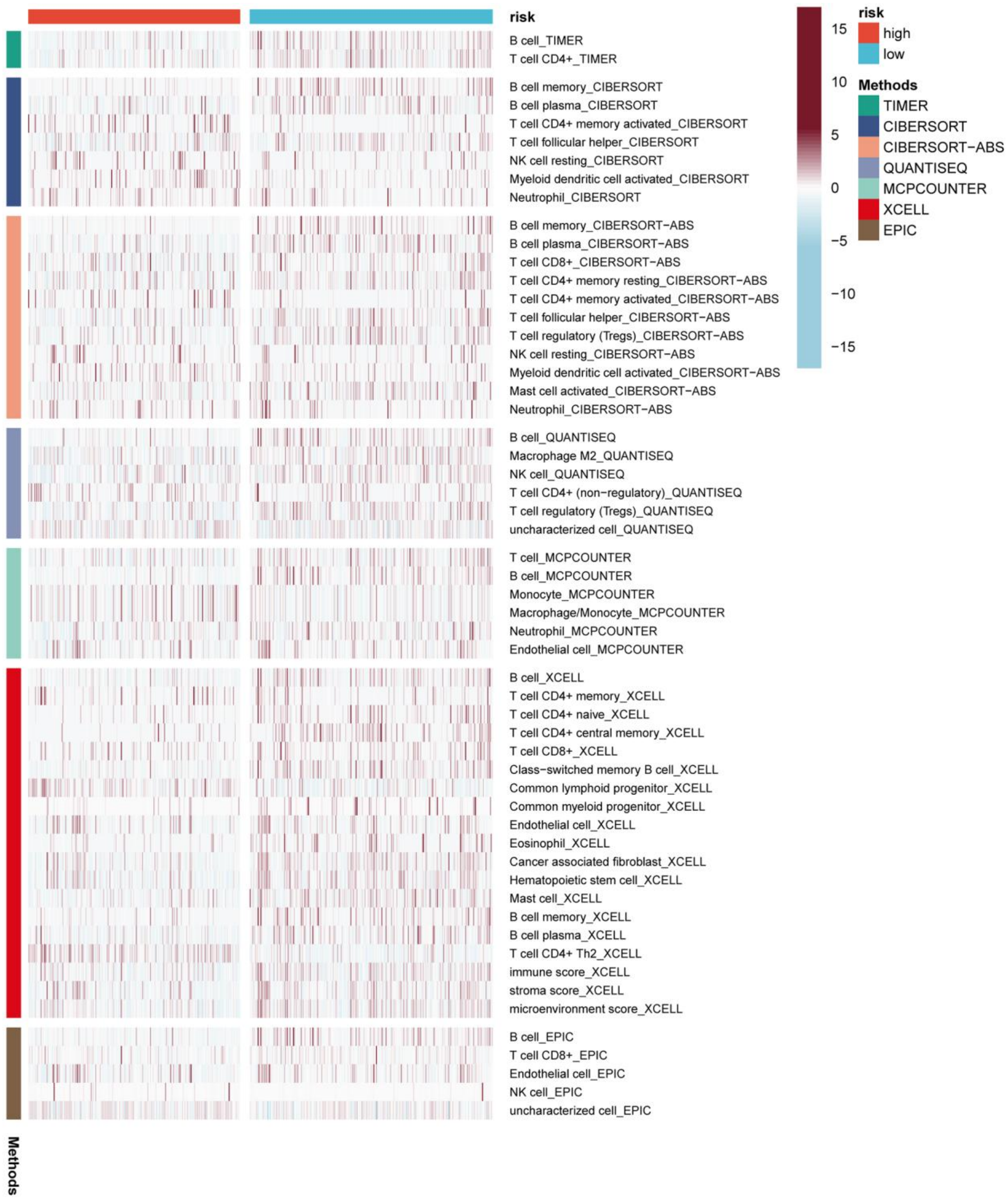

Supplementary Figure 3. Heat map demonstrating the differences in immune cell infiltration between high- and low-risk groups assessed using seven algorithms.
